# Neighborhood deprivation and oncotype DX recurrence scores among Black and White women: the interplay between race, place, and tumor biology

**DOI:** 10.1038/s41523-026-00978-1

**Published:** 2026-05-30

**Authors:** Jasmine M. Miller-Kleinhenz, Lauren E. Barber, Maret L. Maliniak, Lindsay J. Collin, Demetria J. Smith-Graziani, Jeffery M. Switchenko, Kevin C. Ward, Lauren E. McCullough

**Affiliations:** 1 Department of Population Health Science, Bower School of Population Health, University of Mississippi Medical Center, Jackson, MS USA; 2 Department of Epidemiology, Rollins School of Public Health, Emory University, Atlanta, GA USA; 3 Department of Hematology and Medical Oncology, School of Medicine, Emory University, Atlanta, GA USA; 4 Department of Biostatistics and Bioinformatics, Emory University Rollins School of Public Health, Atlanta, GA USA

**Keywords:** Cancer, Diseases, Medical research, Oncology, Risk factors

## Abstract

Breast cancer mortality disparities have persisted between non-Hispanic Black (NHB) and non-Hispanic White (NHW) women, even among those with prognostically favorable disease. The Oncotype DX Recurrence Score (ODX RS) is a gene expression panel used to identify estrogen receptor (ER)-positive breast cancers with aggressive tumor biology, and prior studies suggest higher scores among Black women and in lower-socioeconomic areas. We examined associations of neighborhood deprivation with ODX RS among 11,377 NHB and NHW women diagnosed with ER-positive breast cancer (stage I-IIIa, ≤3 lymph nodes) between 2010-2017 in the Georgia Cancer Registry. The Neighborhood Deprivation Index (NDI), derived via principal component analysis of American Community Survey block-group data, was assessed in quartiles. Prevalence ratios (PR) and 95% confidence intervals (CI) were estimated for high-risk (≥31) versus low-risk (<18) ODX RS. Living in the most deprived neighborhoods was modestly associated with high-risk ODX RS overall (PR = 1.08, 95% CI 0.90–1.30) and among NHW women (PR = 1.12, 95% CI 0.87–1.44), but not among NHB women (PR = 1.01, 95% CI 0.77–1.33). NHB women in the least deprived neighborhoods had the highest prevalence of high-risk ODX RS relative to NHW women in those neighborhoods (PR = 1.92, 95% CI 1.53–2.41). These findings suggest neighborhood deprivation does not explain racial differences in genomic risk.

## Introduction

Breast cancer mortality disparities have persisted between Black and White women for over 30 years^1^. Despite having similar screening rates and initial receipt of guideline-concordant care, Black women are 38% more likely to die than White women^2,3^. Even among prognostically favorable hormone receptor (HR)-positive, human epidermal growth factor receptor-2 (HER2)- negative breast cancers, the Black-White mortality disparity persists, and is more pronounced than among prognostically unfavorable tumors^4^.

There is accumulating evidence that social determinants of health contribute to racial disparities in breast cancer outcomes^5,6^. Neighborhoods are a critical social determinant of health, given that within neighborhoods, residents are exposed to various socioeconomic, psychosocial, physical, and environmental factors that can profoundly impact their health^7,8^. Notably, Black Americans are more likely than their White counterparts to live in resource-deprived, low-socioeconomic neighborhoods^9^. Emerging data suggest that neighborhoods may also play a role in tumor biology^10^. The Oncotype Dx Recurrence Score (ODX RS) is a 21-gene expression panel utilized to identify HR+/HER2− breast cancers that are at a higher risk for recurrence, and may benefit from adjuvant chemotherapy^11–13^. The ODX RS can also be used as a surrogate for tumor biology, with a higher ODX RS indicating a more aggressive tumor biology^14^. Previous studies have found that Black women are more likely to have higher ODX RS than White women^15–17^, and socioeconomic factors (e.g., area-level income, urbanicity) are associated with higher ODX RS^18,19^. There remains a gap in understanding how other neighborhood characteristics, in particular neighborhood deprivation, impact tumor biology in prognostically favorable HR+/HER2− breast cancers, contributing to breast cancer racial disparities.

Limited studies have examined the effects of area-level characteristics on ODX RS^19^. To our knowledge, no study has investigated the association between neighborhood deprivation and ODX RS. Using the neighborhood deprivation index (NDI), a composite measure of neighborhood domains, our goal was to estimate the association between neighborhood deprivation and ODX RS among Black and White women diagnosed with breast cancer in Georgia. In addition, we explored the association between neighborhood deprivation and ODX RS in race-stratified models and the joint effect of neighborhood deprivation and race on ODX RS to help elucidate the interplay between race, place, and tumor biology.

## Results

### Patient and neighborhood characteristics

Of the 11,377 women included in our study, 2777 women (24%) were NHB, and 8600 (76%) were NHW; 4391 (38.6%) patients resided in the least deprived neighborhoods (NDI Quartile 1), and 1514 (13.3%) resided in the most deprived neighborhoods (NDI Quartile 4) (Table 1). Compared with women living in the lowest quartile of NDI, those living in the highest quartile were more likely to have high-risk ODX RS, be ≥50 years of age at diagnosis, and have Medicare or Medicaid insurance at the time of diagnosis. Women residing in the highest quartile were also more likely to live in rural communities, in areas with a majority percentage of Black residents, and in areas with persistent poverty (Table 1). Patient characteristics stratified by both race and NDI quartile are presented in Supplementary Table 2.

**Table 1:** Patient demographic, clinicopathological, and treatment characteristics among 11,377 non-Hispanic Black (NHB) and White (NHW) women diagnosed with stage I-III breast cancer in Georgia with Oncotype Dx Recurrence Scores (ODX) and registered with the Georgia Cancer Registry

Characteristic	Overall, *N* = 11,377	Q1 (Least deprived), *N* = 4391	Q2, *N* = 3099	Q3, *N* = 2373	Q4 (Most deprived), *N* = 1514
Age at diagnosis					
Mean (SD)	59 (11)	58 (11)	59 (11)	60 (11)	60 (11)
Median (IQR)	60 (51, 67)	59 (50, 66)	60 (51, 67)	61 (52, 67)	62 (54, 68)
Age group, *n* (%)					
<50	2334 (21%)	1009 (23%)	662 (21%)	436 (18%)	227 (15%)
50+	9043 (79%)	3382 (77%)	2437 (79%)	1937 (82%)	1287 (85%)
Race/ethnicity, *n* (%)					
Non-Hispanic White	8600 (76%)	3897 (89%)	2359 (76%)	1594 (67%)	750 (50%)
Non-Hispanic Black	2777 (24%)	494 (11%)	740 (24%)	779 (33%)	764 (50%)
ODX recurrence score					
Mean (SD)	18 (11)	18 (10)	18 (11)	18 (11)	18 (12)
Median (IQR)	16 (11, 22)	16 (11, 21)	16 (11, 22)	16 (11, 22)	16 (11, 22)
ODX RS group, *n* (%)					
Low risk	6551 (58%)	2542 (58%)	1773 (57%)	1354 (57%)	882 (58%)
Intermediate risk	3723 (33%)	1491 (34%)	988 (32%)	789 (33%)	455 (30%)
High risk	1103 (9.7%)	358 (8.2%)	338 (11%)	230 (9.7%)	177 (12%)
Stage, *n* (%)					
Stage 1	7811 (69%)	3079 (70%)	2096 (68%)	1612 (68%)	1024 (68%)
Stage 2	3487 (31%)	1273 (29%)	983 (32%)	751 (32%)	480 (32%)
Stage 3	79 (0.7%)	39 (0.9%)	20 (0.6%)	10 (0.4%)	10 (0.7%)
Grade, *n* (%)					
Grade I	3478 (31%)	1445 (33%)	897 (29%)	681 (29%)	455 (31%)
Grade II	5872 (52%)	2239 (52%)	1641 (54%)	1227 (52%)	765 (51%)
Grade III	1892 (17%)	655 (15%)	526 (17%)	441 (19%)	270 (18%)
Unknown	135	52	35	24	24
Lymph nodes, *n* (%)					
LN-	9555 (84%)	3674 (84%)	2585 (83%)	2005 (84%)	1291 (85%)
LN+	1822 (16%)	717 (16%)	514 (17%)	368 (16%)	223 (15%)
Marital status, *n* (%)					
Not married	4093 (37%)	1192 (28%)	1073 (36%)	1027 (45%)	801 (55%)
Married	6859 (63%)	3081 (72%)	1885 (64%)	1247 (55%)	646 (45%)
Insurance type, *n* (%)					
No insurance	145 (1.3%)	33 (0.8%)	49 (1.6%)	34 (1.5%)	29 (2.0%)
Private insurance	5917 (53%)	2639 (61%)	1600 (52%)	1087 (46%)	591 (40%)
Medicaid	611 (5.4%)	97 (2.2%)	143 (4.7%)	186 (7.9%)	185 (12%)
Medicare	3647 (32%)	1211 (28%)	1002 (33%)	841 (36%)	593 (40%)
Other	908 (8.1%)	370 (8.5%)	256 (8.4%)	196 (8.4%)	86 (5.8%)
% of Black residents: ≥50%, *n* (%)	2329 (20%)	190 (4.3%)	519 (17%)	715 (30%)	905 (60%)
% living in persistent poverty, *n* (%)	954 (8.4%)	32 (0.7%)	121 (3.9%)	276 (12%)	525 (35%)
Urban/rural status, *n* (%)					
Urban	8730 (77%)	4012 (91%)	2293 (74%)	1439 (61%)	986 (65%)
Rural	2647 (23%)	379 (8.6%)	806 (26%)	934 (39%)	528 (35%)

Unknown values excluded from percentage calculations.

### Neighborhood deprivation and oncotype DX recurrence scores

In multivariable-adjusted models, living in the highest quartile of NDI was associated with a higher prevalence of high-risk vs low-risk ODX RS (NDI Quartile 4 vs. 1, PR 1.08; 95% CI 0.90, 1.30) (Table 2). In our stratified analyses, we observed little difference in associations by race-ethnicity. In multivariable-adjusted models, living in the highest quartile of NDI was associated with a higher prevalence of high-risk vs low-risk ODX RS among NHW women (Quartile 4 vs. 1, PR 1.12; 95% CI 0.87, 1.44) (Table 3). Among NHB women, we did not observe an association between NDI and ODX RS (Quartile 4 vs. 1, PR 1.01; 95% CI 0.77, 1.33).

**Table 2:** Age- and multivariable-adjusted prevalence ratios (PR) for the association between neighborhood deprivation and ODX Score of Intermediate-risk vs Low-risk and High-risk vs. Low-risk for each quartile of neighborhood deprivation

Neighborhood deprivation index	Low-risk ODX score *N* (%)	Intermediate-risk ODX score *N* (%)	Age-adjusted PR (95% CI)	MV-adjusted PR (95% CI)^a^	High-risk ODX Score *N* (%)	Age-adjusted PR (95% CI)	MV-adjusted PR (95% CI)^a^
Quartile 1 (Least deprived)	2542 (58%)	1491 (34%)	Referent	Referent	358 (8%)	Referent	Referent
Quartile 2	1773 (57%)	988 (32%)	0.97 (0.91, 1.04)	0.97 (0.90, 1.03)	338 (11%)	1.31 (1.15, 1.51)	1.18 (1.03, 1.36)
Quartile 3	1354 (57%)	789 (33%)	1.00 (0.94, 1.08)	0.99 (0.92, 1.06)	230 (10%)	1.20 (1.03, 1.40)	1.00 (0.85, 1.18)
Quartile 4 (Most deprived)	882 (58%)	455 (30%)	0.93 (0.86, 1.01)	0.90 (0.82, 0.99)	177 (12%)	1.39 (1.18, 1.64)	1.08 (0.90, 1.30)

*ODX* oncotype DX, *PR* prevalence ratio, *CI* confidence interval, *MV* multivariable.

^a^Age, race, stage, insurance status, and urban/rural status.

**Table 3:** Age- and multivariable-adjusted prevalence ratios for the association between neighborhood deprivation and ODX score, according to race

Neighborhood deprivation index	Low-risk ODX score *N* (%)	Intermediate-risk ODX score *N* (%)	Age-adjusted PR (95% CI)	MV-adjusted PR (95% CI)^a^	High-risk ODX Score N (%)	Age-adjusted PR (95% CI)	MV-adjusted PR (95% CI)^a^
**Non-Hispanic Black patients**							
Quartile 1 (Least deprived)	250 (51%)	172 (35%)	Referent	Referent	72 (15%)	Referent	Referent
Quartile 2	378 (51%)	251 (34%)	0.98 (0.85, 1.14)	1.00 (0.86, 1.16)	111 (15%)	1.00 (0.77, 1.30)	1.02 (0.79, 1.32)
Quartile 3	418 (54%)	256 (33%)	0.95 (0.82, 1.10)	0.94 (0.81, 1.10)	105 (13%)	0.91 (0.70, 1.19)	0.94 (0.72, 1.23)
Quartile 4 (Most deprived)	411 (54%)	252 (33%)	0.97 (0.83, 1.12)	0.98 (0.84, 1.15)	101 (13%)	0.94 (0.72, 1.23)	1.01 (0.77, 1.33)
**Non-Hispanic White patients**							
Quartile 1 (Least deprived)	2292 (59%)	1319 (34%)	Referent	Referent	286 (7%)	Referent	Referent
Quartile 2	1395 (59%)	737 (31%)	0.95 (0.88, 1.02)	0.95 (0.88, 1.03)	227 (10%)	1.27 (1.08, 1.50)	1.19 (1.01, 1.40)
Quartile 3	936 (59%)	533 (33%)	1.00 (0.92, 1.08)	1.01 (0.93, 1.10)	125 (8%)	1.07 (0.88, 1.31)	0.96 (0.77, 1.19)
Quartile 4 (Most deprived)	471 (63%)	203 (27%)	0.83 (0.74, 0.94)	0.83 (0.73, 0.95)	76 (10%)	1.26 (1.00, 1.60)	1.12 (0.87, 1.44)

*ODX* oncotype DX, *PR* prevalence ratio, *CI* confidence interval, *MV* multivariable.

^a^Age, stage, insurance status, and urban/rural status.

### Stratified analyses by age and stage

When we examined these associations by age, we observed a modest association between neighborhood deprivation and high-risk vs. low-risk ODX RS for both women <50 years of age (Quartile 4, PR 1.04; 95% CI 0.71, 1.54) and ≥50 years of age at diagnosis (Quartile 4, PR 1.09; 95% CI 0.89, 1.34) (Supplementary Table 3). In stage-stratified analyses, we found that neighborhood deprivation was associated with high-risk ODX RS for Stage II–III (Quartile 4, PR 1.27; 95% CI 0.98, 1.65) (Supplementary Table 4). Among women with Stage I, no association was observed.

### Joint effects of race and neighborhood deprivation

To assess the joint effects of neighborhood deprivation and race on ODX RS, we compared each race and NDI quartile with NHW women living in the least deprived neighborhoods (NDI Quartile 1) as the referent group. Among NHW women, there was a non-monotonic increase in the prevalence of high-risk ODX RS as neighborhood deprivation increased with the highest prevalence of high-risk ODX RS in NDI Quartile 2 and Quartile 4 (Quartile 2, PR 1.24; 95% CI 1.05, 1.46; Quartile 3, PR 1.04; 95% CI 0.84, 1.28; Quartile 4, PR 1.23; 95% CI 0.96, 1.56) (Table 4). Among NHB women, we observed a higher prevalence of high-risk ODX RS compared with NHW women at every NDI quartile with the highest prevalence of high-risk ODX RS among NHB women living in the least deprived neighborhoods (Quartile 1, PR 1.92; 95% CI 1.53, 2.41; Quartile 2, PR 1.92; 95% CI 1.57, 2.34; Quartile 3, PR 1.68; 95% CI 1.36, 2.06; Quartile 4, PR 1.70; 95% CI 1.38, 2.10).

**Table 4:** Age- and multivariable-adjusted prevalence ratios for the association between neighborhood deprivation and ODX score, according to race, using the common referent approach with non-Hispanic White and Q1 (least deprived) serving as the referent

Race x NDI	Low-risk ODX score *N* (%)	High-risk ODX score *N* (%)	Age-adjusted PR (95% CI)	MV-adjusted PR (95% CI)^a^
NHW & Q1	2292 (89%)	286 (11%)	Referent	Referent
NHW & Q2	1395 (86%)	227 (14%)	1.29 (1.09, 1.52)	1.24 (1.05, 1.46)
NHW & Q3	936 (88%)	125 (12%)	1.09 (0.90, 1.33)	1.04 (0.84, 1.28)
NHW & Q4	471 (86%)	76 (14%)	1.29 (1.02, 1.64)	1.23 (0.96, 1.56)
NHB & Q1	250 (78%)	72 (22%)	1.98 (1.57, 2.49)	1.92 (1.53, 2.41)
NHB & Q2	378 (77%)	111 (23%)	1.99 (1.64, 2.42)	1.92 (1.57, 2.34)
NHB & Q3	418 (80%)	105 (20%)	1.79 (1.46, 2.19)	1.68 (1.36, 2.06)
NHB & Q4	411 (80%)	101 (20%)	1.80 (1.46, 2.20)	1.70 (1.38, 2.10)

*ODX* oncotype DX, *PR* prevalence ratio, *CI* confidence interval, *MV* multivariable.

^a^Age, stage, insurance status, and urban/rural status. Percentages in Table 4 are calculated among low- and high-risk groups only (excluding intermediate-risk).

### Individual neighborhood indicators by race

To provide more insight into the role of neighborhood in ODX RS, we examined the association between the 8 individual neighborhood indicators comprising the NDI with ODX RS, overall and according to race. We focused on ODX RS low-risk and high-risk scores. In multivariable-adjusted models, for every 10-unit increase in the percentage (%) of a neighborhood indicator, the prevalence of high-risk ODX RS (vs. low-risk ODX RS) was determined. Among NHW women, we found a lower prevalence of high-risk ODX RS for % of individuals employed in managerial or administrative jobs (PR 0.91; 95% CI 0.83, 1.00) (Table 5). Among NHB women, we observed a higher prevalence of high-risk ODX RS for % of individuals employed in managerial or administrative jobs (PR 1.08; 95% CI 0.93, 1.24) (Table 5). The remaining neighborhood indicators’ associations were modest or near null.

**Table 5:** Age- and multivariable-adjusted prevalence ratios for the association between neighborhood characteristics and ODX score (10-unit increase) overall and by race

	Overall		NHW		NHB	
Neighborhood deprivation index components (Continuous)	Age-adjusted PR (95% CI)	MV-adjusted PR (95% CI)^a^	Age-adjusted PR (95% CI)	MV-adjusted PR (95% CI)^a^	Age-adjusted PR (95% CI)	MV-adjusted PR (95% CI)^a^
% of individuals with annual incomes below the federal poverty line	1.03 (0.99, 1.07)	0.97 (0.93, 1.01)	1.00 (0.94, 1.06)	0.96 (0.91, 1.03)	0.96 (0.90, 1.02)	0.98 (0.92, 1.04)
% of households receiving public assistance	1.09 (0.90, 1.34)	0.96 (0.78, 1.18)	1.02 (0.78, 1.33)	0.93 (0.71, 1.23)	0.96 (0.70, 1.33)	0.97 (0.71, 1.33)
% of female-headed households with children younger than 18 years	1.13 (1.06, 1.20)	1.03 (0.96, 1.10)	1.07 (0.97, 1.18)	1.06 (0.96, 1.17)	0.98 (0.89, 1.08)	0.99 (0.90, 1.09)
% of households with annual income less than $35,000	1.04 (1.01, 1.07)	0.98 (0.95, 1.02)	1.01 (0.97, 1.05)	0.97 (0.93, 1.02)	0.98 (0.94, 1.02)	1.00 (0.95, 1.04)
% of individuals employed in managerial or administrative jobs	0.86 (0.80, 0.93)	0.94 (0.87, 1.02)	0.87 (0.79, 0.96)	0.91 (0.83, 1.00)	1.09 (0.95, 1.25)	1.08 (0.93, 1.24)
% of households with more than 1 person per room	1.16 (0.99, 1.35)	1.05 (0.90, 1.23)	1.07 (0.87, 1.31)	1.04 (0.84, 1.28)	1.10 (0.86, 1.40)	1.07 (0.84, 1.36)
% of unemployed individuals	1.16 (1.08, 1.25)	1.05 (0.97, 1.14)	1.06 (0.95, 1.19)	1.04 (0.93, 1.17)	1.03 (0.92, 1.16)	1.05 (0.94, 1.17)
% of individuals aged 25 years or older without a high school degree or General Educational Development credentials	1.06 (1.00, 1.12)	1.00 (0.95, 1.07)	1.06 (0.99, 1.13)	1.01 (0.93, 1.10)	0.97 (0.89, 1.07)	1.00 (0.91, 1.11)

*ODX* oncotype DX, *PR* prevalence ratio, *CI* confidence interval, *MV* multivariable.

^a^Age, race (for overall model only), stage, insurance status, and urban/rural status.

## Discussion

In this study of NHB and NHW breast cancer patients, we observed only modest differences in the association between neighborhood deprivation and the prevalence of high-risk ODX RS overall and by race. Overall, higher neighborhood deprivation was associated with a slightly higher prevalence of high-risk scores, although estimates were imprecise. In race-stratified analyses, a similar pattern was observed among NHW women, but these estimates were also imprecise. Among NHB women, no clear association between neighborhood deprivation and the prevalence of high-risk ODX RS was observed. Notably, NHB women residing in the least deprived neighborhoods had among the highest prevalence of high-risk scores. To further explore these patterns, we examined individual neighborhood indicators. We did not observe consistent patterns across domains, and most associations were modest and imprecise. The only notable pattern was observed for the occupational indicator representing the percentage of individuals employed in managerial or administrative roles. In overall analyses and among NHW women, higher representation in these occupations was associated with a lower prevalence of high-risk ODX RS, whereas the direction of association was reversed among NHB women, which may warrant further investigation. Overall, there was little evidence that individual neighborhood components were consistently associated with recurrence score, particularly among NHB women.

Neighborhoods are an important determinant of health, with research indicating that health outcomes are often more driven by ZIP code than genetic code^20^. Racial minorities are more likely to live in neighborhoods that are considered disadvantaged or deprived, which may contribute to the observed racial disparities in health outcomes. Many recent studies of neighborhood deprivation have examined its impact on breast cancer mortality, reporting an association between neighborhood deprivation and increased breast cancer mortality^21–23^. Some have shown that there are distinct differences by race^21,23^. For example, a study by Barber et al. found that NHW women living in deprived neighborhoods had a higher risk of breast cancer mortality, but this association was not observed among NHB women^21^. Few studies have investigated the association between neighborhood deprivation and ODX RS^19^. One study in the National Cancer Database found that living in a disadvantaged neighborhood, measured by having an annual neighborhood-level income of less than $48,000, was associated with a slightly higher odds of high-risk ODX RS compared with living in an advantaged neighborhood, after adjustment for sociodemographic factors and stage (OR 1.04, 95% CI 1.01, 1.07). This estimate was similar to our overall multivariable-adjusted result using the same TAILORx cut-points (Q4 vs Q1: PR 1.05, 95% CI 0.92, 1.21)^19^. However, since our study used quartiles of neighborhood deprivation rather than a binary exposure, we were able to assess dose-response patterns and found no clear gradient in high-risk ODX RS across increasing levels of deprivation. We also found that NHB women had a higher proportion of high-risk ODX RS^15,17^, which aligns with prior research and informed our approach to investigate the interplay of race, neighborhood, and ODX RS. To our knowledge, no other studies have examined whether the association between neighborhood deprivation and ODX RS differs by race. In this study, we did not observe a consistent overall association, and while race-stratified analyses suggested potential heterogeneity for % of individuals employed in managerial or administrative jobs, estimates were imprecise and warrant cautious interpretation.

Emerging research indicates that living in low-socioeconomic neighborhoods is associated with more aggressive tumor biology^24–26^. One study found that living in deprived neighborhoods was associated with the up-regulation of genes related to tumor aggressiveness, activation of the sympathetic nervous system, and a “fight or flight” stress response^22^. The up-regulation of pro-inflammatory genes was also found to be associated with higher ODX RS. Our results are directionally consistent with the findings of these studies, at least for NHW women. NHB women had higher ODX RS at every neighborhood quartile. Black women might experience sustained and cumulative exposure to adverse social, physical, psychological, and chemical stressors within their residential, educational, employment, and other environments, resulting in biological weathering and poorer health outcomes^27–29^. Interestingly, we observed that NHB women living in the least deprived neighborhoods have the highest prevalence of high-risk ODX RS compared with NHW women who live in the least deprived neighborhoods. These results underscore that NHB women may experience diminishing health gains compared to their NHW counterparts in resource-rich socioeconomic environments^30^. Furthermore, NHB women may experience adverse psychosocial stressors in addition to the neighborhood exposures that affect biological processes and potentially tumor biology and aggressiveness. These early findings indicate that NHB women may have more aggressive tumors, irrespective of neighborhood, and poorer outcomes, even with similar ODX RS as White women^15,31^. The collective data to date may suggest that we consider whether ODX RS is the most clinically relevant tool for determining treatment and risk of recurrence in this population. Future work should include more robust genomic panels to elucidate the biological differences that contribute to disparities in outcomes.

There are limitations to this study that should be considered. Our analysis was limited to women with an available ODX RS in the Georgia Cancer Registry between 2010 and 2017, potentially introducing selection bias. In addition, the use of registry data did not provide information on which clinical decisions led to some patients not receiving ODX RS testing or whether they received alternative genomic testing. Although differences in ODX availability by race and neighborhood deprivation were modest, our analytic sample may underrepresent patients at the highest socioeconomic disadvantage, potentially attenuating observed associations between neighborhood deprivation and ODX RS. Additionally, differential mammography screening by neighborhood deprivation could introduce detection bias, with less deprived women more likely to have screen-detected, lower-risk tumors, although this concern is likely limited in our study given the restriction to early-stage (predominantly stages I–II) disease, which narrows the range of tumor characteristics across deprivation groups. NDI is a multidimensional index that only focuses on the socioeconomic context of a neighborhood. While other individual and neighborhood factors could influence tumor biology, including pollution, nutritional landscape, and the social and built environments, these factors were not accounted for in the present study. We measured neighborhood deprivation based on a woman’s address at diagnosis, but we were not able to assess residential history prior to diagnosis. While it is important to understand how neighborhood-level exposures impact health over the life-course, it is unlikely that a change in residence would result in robust misclassification, as patients are likely to have moved from a similar SES neighborhood. Lastly, although Georgia is a racially, socioeconomically, and geographically diverse population with known racial disparities in breast cancer outcomes, our findings may have limited generalizability in regions outside of Georgia.

In this study, we found that neighborhood deprivation was associated with a slightly higher prevalence of high-risk ODX RS among NHW women but not among NHB women, although the pattern among NHW women was non-monotonic and imprecise. Across all levels of neighborhood deprivation, NHB women had a higher prevalence of high-risk ODX RS than NHW women. These findings suggest that neighborhood deprivation does not explain the higher prevalence of aggressive tumor biology, as captured by ODX RS, among NHB women. While neighborhood deprivation may still contribute to variation in genomic risk among NHW women, larger studies are needed to confirm this association. Our findings substantiate that Black women may be impacted by their neighborhoods in different ways than NHW women and that other exposures and stressors likely contribute to persistent disparities in tumor biology associated with poor prognosis. The intersection between race, neighborhood, and tumor biology requires a more nuanced investigation of the social-structural drivers of disparate breast cancer outcomes. Further research is needed to identify intervenable factors within and beyond the neighborhood that are driving racial disparities in breast cancer outcomes.

## Methods

### Study population

Using the Georgia Cancer Registry (GCR), a statewide population-based registry, we identified Non-Hispanic Black (NHB) and Non-Hispanic White (NHW) women diagnosed with a first primary breast cancer (International Classification of Diseases for Oncology, 3rd edition, ICD-O-3 = C50) occurring between 2010 and 2017 (*N* = 53,264). We restricted our patient population to women ≥18 years old and considered eligible to receive Oncotype DX testing—Stage I-III patients with HR+/HER2− disease and either node-negative (N0) or with limited lymph node involvement (N1) (*N* = 25,771). Patients were further excluded if they were missing neighborhood deprivation (*n* = 18) or ODX RS (*N* = 14,376), yielding a final analytic sample of 11,377 women (Supplementary Fig. 1). Compared to included women, those considered eligible but missing ODX RS were generally older at diagnosis, later stage, more likely to be node-positive, and less likely to be married or have private insurance (Supplementary Table 1). Differences by neighborhood deprivation and race were modest: the proportion living in the most deprived neighborhoods was similar among women with and without ODX data (13% vs. 16%), as was the proportion who were NHB (24% vs. 27%). The pattern of missingness was similar by race. Notably, ODX RS availability increased over the study period for both NHB and NHW women. This study was performed in accordance with the principles of the Declaration of Helsinki. The study protocol was reviewed and approved by the Emory University Institutional Review Board (IRB #00099875). Because the analysis used de-identified secondary data obtained from the Georgia Cancer Registry, the requirement for informed consent was waived by the Emory University Institutional Review Board.

### Neighborhood deprivation index

Neighborhood deprivation served as the primary exposure of interest and was quantified using the NDI developed by Messer and colleagues. The methodology for constructing the NDI has been detailed previously^21,32^. We calculated an NDI score for each block group in Georgia by applying principal component analysis to block group-level data from the 2011–2015 American Community Survey (ACS). Eight socioeconomic indicators were included in this analysis: the proportion of individuals living below the poverty line, proportion of households receiving public assistance, proportion of female-headed households with children under 18 years, proportion of households earning less than $35,000, proportion of unemployed individuals, proportion of individuals employed in managerial or administrative positions, proportion of households with more than one person per room, and proportion of individuals aged 25 or older without a high school diploma or General Educational Development credentials (GED). Census block groups were chosen as the geographic unit of analysis, as we considered them an approximate representation of neighborhoods. Following principal component analysis, we assigned weights to each indicator using the factor loadings from the first principal component. The weighted indicators were then summed to generate an NDI score, which was divided into quartiles based on the statewide distribution in Georgia. The highest quartile corresponded to neighborhoods experiencing the greatest neighborhood deprivation. To integrate NDI values with patient data, we linked the scores to the block group that corresponded to patients’ addresses at the time of diagnosis, which was geocoded by the GCR.

### Oncotype DX recurrence score

Results from the 21-gene ODX RS assay (Exact Sciences, Corp., Madison, WI, USA) are available to the GCR as a component of the National Cancer Institute’s Surveillance, Epidemiology, and End Results (SEER) Program through a direct linkage with Exact Sciences and through manual collection of multigene signature testing and results by the GCR (Site Specific Factors 22 and 23)^33^. We used ODX RS from the Exact Sciences linkage when available (*N* = 11,029) and only used the ODX RS from manual abstraction when there was no score from Exact Sciences (*N* = 348). The guidelines categorizing the ODX RS into low-risk [<18], intermediate-risk [18–30], and high-risk [≥31] groups were used in this analysis as they reflect the standard scores utilized in clinical practice at the time of diagnosis (2010–2017)^34^. The low-risk group served as the reference against which high scores were compared^35^.

We conducted sensitivity analyses utilizing the cut-points from the TAILORx trial, high-risk [≥26] and low-risk [0–25]^36^. Estimates were similar to our primary results; therefore, we present only the primary analyses. Results from TAILORx cut points are presented in the supplement (Supplementary Table 5 for the distribution of TAILORx risk groups, Supplementary Table 6 for the overall association between neighborhood deprivation and TAILORx high-risk vs low-risk ODX RS, and Supplementary Table 7 for race-stratified estimates).

### Patient characteristics

Race and ethnicity were obtained by the GCR using medical records and other information from the reporting facility^37^. Other variables of interest available in the GCR included age at diagnosis, marital status (e.g., married [common law and unmarried domestic] or other [single, divorced, widowed, or separated]), and insurance status at diagnosis (private, Medicare, Medicaid, uninsured, or other).

### Neighborhood characteristics

Neighborhood characteristics—including the proportion of the population identified as Black (≥50% or <50%) and persistent poverty status (≥20% of the population living below the federal poverty level for the 30-year period from 1990 to 2015–2019)—were derived using data from the census and ACS. The proportion of the population identified as Black was measured at the block group level, while persistent poverty was measured at the census track level. Data on county-level rurality was derived by the Georgia Department of Public Health.

### Statistical analysis

We calculated descriptive statistics for covariates overall and across NDI quartiles. Modified Poisson regression with robust variance estimates was used to compute age- and multivariable-adjusted prevalence ratios (PRs) and 95% confidence intervals (CIs) for the association between quartiles of NDI and ODX RS groups^38^. We conducted analyses comparing intermediate-risk and low-risk scores, as well as high-risk and low-risk scores. For our intermediate-risk analyses, the results were either null or modest; therefore, we focused on reporting the results from our high-risk analyses. Age, race, stage, insurance status, and county-level rurality were identified *a* priori as confounders, based on previous literature and graphical-based methods^39^.

The distribution of NDI is differential by race, and may impact NHB and NHW breast cancer patients differently; therefore, we conducted race-stratified (NHB and NHW) analyses between neighborhood deprivation and ODX RS. We conducted two additional stratified analyses to further investigate the relationship between neighborhood deprivation, ODX RS, and clinically relevant factors for determining treatment recommendations. First, we conducted age-stratified analyses. Age and menopausal status are often taken into consideration when determining the course of treatment, in addition to the ODX RS score. While we do not have menopausal status, our age-stratified analyses were dichotomized at age 50 as a proxy for menopausal status (<50 and ≥50 years of age), as pre-menopausal women during the time of this study were more likely to get chemotherapy regardless of their Oncotype DX RS^36^. Next, we conducted stage-stratified analyses (Stage I vs. stages II–III) because stage and lymph node involvement are determining factors for adjuvant chemotherapy use in HR+/HER2− breast cancer. Additionally, we investigated the association between individual neighborhood indicators and ODX RS by race.

All analyses were performed using SAS, version 9.4 (SAS Institute Inc.).

## Supplementary information

Supplementary Information

## Data Availability

The datasets generated and/or analyzed during the current study are not publicly available because they contain protected health information from the Georgia Cancer Registry and are subject to data use agreements and IRB-approved protocols restricting redistribution, but are available from the senior author on reasonable request and with appropriate regulatory approvals (lauren.mccullough@emory.edu).
