# Supplementary material for: Neighborhood deprivation and oncotype DX recurrence scores among Black and White women: the interplay between race, place, and tumor biology
Source: NPJ Breast Cancer. 2026 May 30;12:111. doi: 10.1038/s41523-026-00978-1 (PMC13519022; doi:10.1038/s41523-026-00978-1)
Supplement: Supplementary file 1 [file 41523_2026_978_MOESM1_ESM.pdf]

Supplementary Materials:

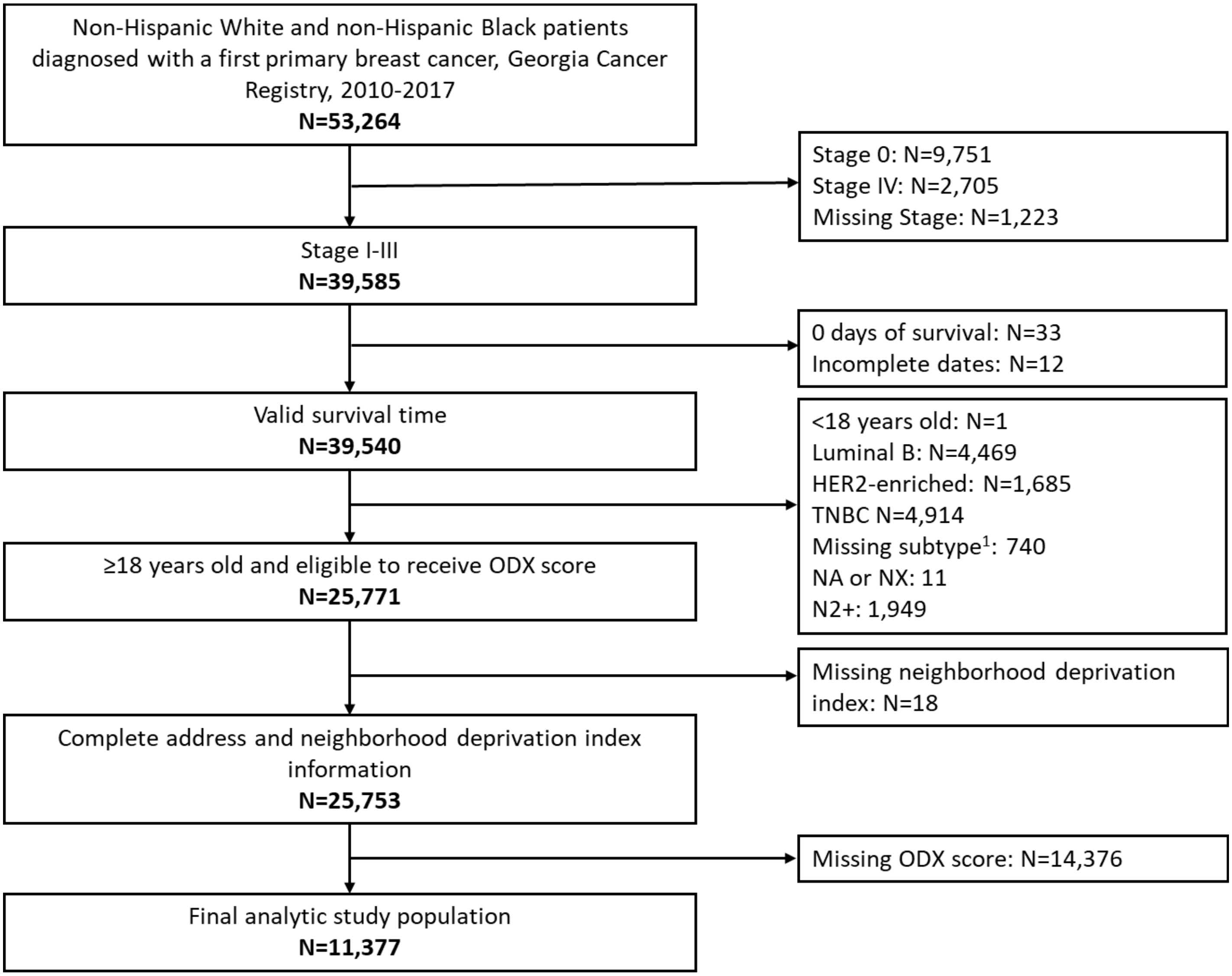

Supplementary Figure 1. Patient selection into the analytic study population.

[1]Because HER2 status availability varied over the study period, ER+ patients with missing or borderline HER2 status were considered eligible to receive ODX score.

Supplementary Table 1. Patient characteristics according to availability of Oncotype DX recurrence score among patients considered eligible for receipt of Oncotype DX testing and not missing neighborhood deprivation index (N=25,753), Georgia Cancer Registry (2010-2017).

Characteristic	Overall		Non-Hispanic White		Non-Hispanic Black	
	**ODX, N = 11,377**	**Missing ODX, N = 14,376**	**ODX, N = 8,600**	**Missing ODX, N = 10,428**	**ODX, N = 2,777**	**Missing ODX, N = 3,948**
Age at diagnosis						
Mean (SD)	59 (11)	64 (14)	60 (11)	65 (14)	57 (11)	60 (15)
Median (IQR)	60 (51, 67)	64 (53, 74)	61 (52, 67)	66 (55, 75)	58 (49, 66)	60 (49, 70)
Age group, n (%)						
<40	398 (3.5%)	651 (4.5%)	247 (2.9%)	336 (3.2%)	151 (5.4%)	315 (8.0%)
40-49	1,936 (17%)	1,946 (14%)	1,388 (16%)	1,225 (12%)	548 (20%)	721 (18%)
50-59	3,174 (28%)	2,842 (20%)	2,356 (27%)	1,924 (18%)	818 (29%)	918 (23%)
60-69	3,930 (35%)	3,696 (26%)	3,053 (36%)	2,750 (26%)	877 (32%)	946 (24%)
70-79	1,772 (16%)	3,227 (22%)	1,415 (16%)	2,579 (25%)	357 (13%)	648 (16%)
80+	167 (1.5%)	2,014 (14%)	141 (1.6%)	1,614 (15%)	26 (0.9%)	400 (10%)
Race/ethnicity, n (%)						
Non-Hispanic White	8,600 (76%)	10,428 (73%)				
Non-Hispanic Black	2,777 (24%)	3,948 (27%)				
Neighborhood deprivation index, n (%)						
Q1 (Least deprived)	4,391 (39%)	4,849 (34%)	3,897 (45%)	4,220 (40%)	494 (18%)	629 (16%)
Q2	3,099 (27%)	4,031 (28%)	2,359 (27%)	3,063 (29%)	740 (27%)	968 (25%)
Q3	2,373 (21%)	3,169 (22%)	1,594 (19%)	2,049 (20%)	779 (28%)	1,120 (28%)
Q4 (Most deprived)	1,514 (13%)	2,327 (16%)	750 (8.7%)	1,096 (11%)	764 (28%)	1,231 (31%)
Stage, n (%)						
Stage 1	7,811 (69%)	7,970 (55%)	6,028 (70%)	6,175 (59%)	1,783 (64%)	1,795 (45%)
Stage 2	3,487 (31%)	5,587 (39%)	2,512 (29%)	3,771 (36%)	975 (35%)	1,816 (46%)
Stage 3	79 (0.7%)	819 (5.7%)	60 (0.7%)	482 (4.6%)	19 (0.7%)	337 (8.5%)
Grade, n (%)						
Grade I	3,478 (31%)	4,388 (32%)	2,789 (33%)	3,493 (35%)	689 (25%)	895 (24%)
Grade II	5,872 (52%)	6,124 (44%)	4,436 (52%)	4,546 (45%)	1,436 (52%)	1,578 (42%)
Grade III	1,892 (17%)	3,254 (24%)	1,277 (15%)	1,978 (20%)	615 (22%)	1,276 (34%)
Unknown	135	610	98	411	37	199
Lymph nodes, n (%)						
LN-	9,555 (84%)	10,079 (70%)	7,233 (84%)	7,597 (73%)	2,322 (84%)	2,482 (63%)
LN+	1,822 (16%)	4,297 (30%)	1,367 (16%)	2,831 (27%)	455 (16%)	1,466 (37%)

Characteristic	Overall		Non-Hispanic White		Non-Hispanic Black	
	ODX, N = 11,377	Missing ODX, N = 14,376	ODX, N = 8,600	Missing ODX, N = 10,428	ODX, N = 2,777	Missing ODX, N = 3,948
Marital status, n (%)						
Not married	4,093 (37%)	6,538 (48%)	2,545 (31%)	4,170 (42%)	1,548 (59%)	2,368 (63%)
Married	6,859 (63%)	7,165 (52%)	5,771 (69%)	5,803 (58%)	1,088 (41%)	1,362 (37%)
Unknown	425	673	284	455	141	218
Insurance type, n (%)						
No insurance	145 (1.3%)	212 (1.5%)	88 (1.0%)	112 (1.1%)	57 (2.1%)	100 (2.6%)
Private insurance	5,917 (53%)	5,545 (39%)	4,558 (54%)	3,935 (38%)	1,359 (50%)	1,610 (41%)
Medicaid	611 (5.4%)	877 (6.2%)	296 (3.5%)	410 (4.0%)	315 (12%)	467 (12%)
Medicare	3,647 (32%)	6,575 (46%)	2,861 (34%)	5,165 (50%)	786 (29%)	1,410 (36%)
Other	908 (8.1%)	938 (6.6%)	687 (8.1%)	627 (6.1%)	221 (8.1%)	311 (8.0%)
Unknown	149	229	110	179	39	50
% of Black residents: >=50%, n (%)	2,329 (20%)	3,123 (22%)	678 (7.9%)	817 (7.8%)	1,651 (59%)	2,306 (58%)
Persistent poverty, n (%)	954 (8.4%)	1,291 (9.0%)	491 (5.7%)	543 (5.2%)	463 (17%)	748 (19%)
Urban/rural status, n (%)						
Urban	8,730 (77%)	10,997 (76%)	6,391 (74%)	7,682 (74%)	2,339 (84%)	3,315 (84%)
Rural	2,647 (23%)	3,379 (24%)	2,209 (26%)	2,746 (26%)	438 (16%)	633 (16%)
Chemotherapy as part of initial treatment, n (%)						
None	8,075 (72%)	8,348 (59%)	6,301 (75%)	6,520 (63%)	1,774 (65%)	1,828 (47%)
Chemotherapy	2,404 (22%)	4,896 (35%)	1,679 (20%)	3,124 (30%)	725 (27%)	1,772 (46%)
Not recommended/died before therapy	64 (0.6%)	237 (1.7%)	48 (0.6%)	163 (1.6%)	16 (0.6%)	74 (1.9%)
Recommended but not administered	608 (5.5%)	671 (4.7%)	413 (4.9%)	478 (4.6%)	195 (7.2%)	193 (5.0%)
Unknown	226	224	159	143	67	81
Year of diagnosis, n (%)						
2010	1,087 (9.6%)	1,798 (13%)	846 (9.8%)	1,341 (13%)	241 (8.7%)	457 (12%)
2011	1,290 (11%)	1,785 (12%)	1,019 (12%)	1,322 (13%)	271 (9.8%)	463 (12%)
2012	1,340 (12%)	1,772 (12%)	1,028 (12%)	1,298 (12%)	312 (11%)	474 (12%)
2013	1,410 (12%)	1,789 (12%)	1,058 (12%)	1,265 (12%)	352 (13%)	524 (13%)
2014	1,439 (13%)	1,776 (12%)	1,067 (12%)	1,293 (12%)	372 (13%)	483 (12%)
2015	1,505 (13%)	1,735 (12%)	1,117 (13%)	1,270 (12%)	388 (14%)	465 (12%)
2016	1,666 (15%)	1,886 (13%)	1,240 (14%)	1,349 (13%)	426 (15%)	537 (14%)
2017	1,640 (14%)	1,835 (13%)	1,225 (14%)	1,290 (12%)	415 (15%)	545 (14%)

Supplementary Table 2. Patient demographic, clinicopathological, and treatment characteristics stratified by race/ethnicity and quartile of the neighborhood deprivation index.

Characteristic	Non-Hispanic White					Non-Hispanic Black				
	All NHW N = 8,600	Q1 (least deprived) N = 3,897	Q2 N = 2,359	Q3 N = 1,594	Q4 (most deprived) N = 750	All NHB N = 2,777	Q1 (least deprived) N = 494	Q2 N = 740	Q3 N = 779	Q4 (most deprived) N = 764
Age at diagnosis										
Mean (SD)	60 (11)	59 (10)	60 (11)	61 (11)	61 (10)	57 (11)	56 (11)	56 (11)	58 (11)	60 (11)
Median (IQR)	61 (52, 67)	59 (50, 66)	61 (52, 68)	62 (54, 68)	62 (55, 69)	58 (49, 66)	56 (47, 64)	56 (48, 64)	58 (50, 65)	60 (52, 67)
Age group, n (%)										
<50	1,635 (19%)	857 (22%)	436 (18%)	249 (16%)	93 (12%)	699 (25%)	152 (31%)	226 (31%)	187 (24%)	134 (18%)
50+	6,965 (81%)	3,040 (78%)	1,923 (82%)	1,345 (84%)	657 (88%)	2,078 (75%)	342 (69%)	514 (69%)	592 (76%)	630 (82%)
ODX recurrence score										
Mean (SD)	17 (10)	17 (10)	17 (11)	17 (10)	17 (11)	19 (12)	20 (13)	20 (13)	19 (12)	19 (12)
Median (IQR)	16 (11, 21)	16 (11, 21)	16 (11, 22)	16 (11, 22)	15 (10, 22)	17 (11, 24)	17 (12, 25)	17 (11, 25)	17 (12, 24)	17 (11, 24)
ODX RS group, n (%)										
Low risk	5,094 (59%)	2,292 (59%)	1,395 (59%)	936 (59%)	471 (63%)	1,457 (52%)	250 (51%)	378 (51%)	418 (54%)	411 (54%)
Intermediate risk	2,792 (32%)	1,319 (34%)	737 (31%)	533 (33%)	203 (27%)	931 (34%)	172 (35%)	251 (34%)	256 (33%)	252 (33%)
High risk	714 (8.3%)	286 (7.3%)	227 (9.6%)	125 (7.8%)	76 (10%)	389 (14%)	72 (15%)	111 (15%)	105 (13%)	101 (13%)
Stage, n (%)										
Stage 0	0 (0%)	0 (0%)	0 (0%)	0 (0%)	0 (0%)	0 (0%)	0 (0%)	0 (0%)	0 (0%)	0 (0%)
Stage 1	6,028 (70%)	2,782 (71%)	1,616 (69%)	1,106 (69%)	524 (70%)	1,783 (64%)	297 (60%)	480 (65%)	506 (65%)	500 (65%)
Stage 2	2,512 (29%)	1,082 (28%)	728 (31%)	482 (30%)	220 (29%)	975 (35%)	191 (39%)	255 (34%)	269 (35%)	260 (34%)
Stage 3	60 (0.7%)	33 (0.8%)	15 (0.6%)	6 (0.4%)	6 (0.8%)	19 (0.7%)	6 (1.2%)	5 (0.7%)	4 (0.5%)	4 (0.5%)
Stage 4	0 (0%)	0 (0%)	0 (0%)	0 (0%)	0 (0%)	0 (0%)	0 (0%)	0 (0%)	0 (0%)	0 (0%)
Grade, n (%)										
Grade I	2,789 (33%)	1,327 (34%)	724 (31%)	491 (31%)	247 (33%)	689 (25%)	118 (24%)	173 (24%)	190 (25%)	208 (28%)

Grade II	4,436 (52%)	1,983 (51%)	1,246 (53%)	828 (52%)	379 (51%)	1,436 (52%)	256 (52%)	395 (54%)	399 (52%)	386 (51%)
Grade III	1,277 (15%)	541 (14%)	364 (16%)	259 (16%)	113 (15%)	615 (22%)	114 (23%)	162 (22%)	182 (24%)	157 (21%)
Lymph nodes, n (%)										
LN-	7,233 (84%)	3,267 (84%)	1,968 (83%)	1,344 (84%)	654 (87%)	2,322 (84%)	407 (82%)	617 (83%)	661 (85%)	637 (83%)
LN+	1,367 (16%)	630 (16%)	391 (17%)	250 (16%)	96 (13%)	455 (16%)	87 (18%)	123 (17%)	118 (15%)	127 (17%)
Marital status, n (%)										
Not married	2,545 (31%)	955 (25%)	713 (32%)	569 (37%)	308 (42%)	1,548 (59%)	237 (50%)	360 (51%)	458 (62%)	493 (68%)
Married	5,771 (69%)	2,846 (75%)	1,540 (68%)	968 (63%)	417 (58%)	1,088 (41%)	235 (50%)	345 (49%)	279 (38%)	229 (32%)
Insurance type, n (%)										
No insurance	88 (1.0%)	28 (0.7%)	31 (1.3%)	17 (1.1%)	12 (1.6%)	57 (2.1%)	5 (1.0%)	18 (2.5%)	17 (2.2%)	17 (2.3%)
Private insurance	4,558 (54%)	2,354 (61%)	1,196 (52%)	715 (45%)	293 (40%)	1,359 (50%)	285 (58%)	404 (55%)	372 (49%)	298 (40%)
Medicaid	296 (3.5%)	65 (1.7%)	79 (3.4%)	86 (5.4%)	66 (9.0%)	315 (12%)	32 (6.5%)	64 (8.8%)	100 (13%)	119 (16%)
Medicare	2,861 (34%)	1,092 (28%)	830 (36%)	620 (39%)	319 (44%)	786 (29%)	119 (24%)	172 (24%)	221 (29%)	274 (36%)
Other	687 (8.1%)	322 (8.3%)	185 (8.0%)	140 (8.9%)	40 (5.5%)	221 (8.1%)	48 (9.8%)	71 (9.7%)	56 (7.3%)	46 (6.1%)
**Area-level characteristics**										
≥50% Black residents, n (%)	678 (7.9%)	76 (2.0%)	135 (5.7%)	201 (13%)	266 (35%)	1,651 (59%)	114 (23%)	384 (52%)	514 (66%)	639 (84%)
% living in persistent poverty, n (%)	491 (5.7%)	31 (0.8%)	84 (3.6%)	185 (12%)	191 (25%)	463 (17%)	1 (0.2%)	37 (5.0%)	91 (12%)	334 (44%)
Urban/rural status, n (%)										
Urban	6,391 (74%)	3,536 (91%)	1,650 (70%)	808 (51%)	397 (53%)	2,339 (84%)	476 (96%)	643 (87%)	631 (81%)	589 (77%)
Rural	2,209 (26%)	361 (9.3%)	709 (30%)	786 (49%)	353 (47%)	438 (16%)	18 (3.6%)	97 (13%)	148 (19%)	175 (23%)

Supplementary Table 3. Age- and multivariable-adjusted prevalence ratios for the association between neighborhood deprivation and ODX score, according to age at diagnosis.

Neighborhood deprivation index	Low-risk ODX Score N (%)	Intermediate-risk ODX Score N (%)	Age-adjusted PR (95% CI)	MV-adjusted PR (95% CI)[a]	High-risk ODX Score N (%)	Age-adjusted PR (95% CI)	MV-adjusted PR (95% CI)[a]
**<50 years old patients**							
Quartile 1 (Least deprived)	562 (56%)	360 (36%)	Referent	Referent	87 (9%)	Referent	Referent
Quartile 2	346 (52%)	228 (34%)	1.01 (0.89, 1.15)	0.97 (0.85, 1.11)	88 (13%)	1.51 (1.15, 1.97)	1.26 (0.96, 1.65)
Quartile 3	230 (53%)	150 (34%)	1.00 (0.87, 1.16)	0.94 (0.80, 1.10)	56 (13%)	1.45 (1.07, 1.97)	1.12 (0.81, 1.55)
Quartile 4 (Most deprived)	126 (56%)	67 (30%)	0.87 (0.70, 1.07)	0.78 (0.62, 0.97)	34 (15%)	1.50 (1.05, 2.13)	1.04 (0.71, 1.54)
**≥ 50 years old patients**							
Quartile 1 (Least deprived)	1980 (59%)	1131 (33%)	Referent	Referent	271 (8%)	Referent	Referent
Quartile 2	1427 (59%)	760 (31%)	0.96 (0.89, 1.03)	0.96 (0.89, 1.04)	250 (10%)	1.25 (1.06, 1.47)	1.15 (0.98, 1.36)
Quartile 3	1124 (58%)	639 (33%)	1.00 (0.93, 1.08)	1.00 (0.92, 1.09)	174 (9%)	1.12 (0.94, 1.34)	0.97 (0.80, 1.17)
Quartile 4 (Most deprived)	756 (59%)	388 (30%)	0.94 (0.85, 1.03)	0.93 (0.84, 1.02)	143 (11%)	1.33 (1.11, 1.61)	1.09 (0.89, 1.34)

ODX, Oncotype DX; PR, Prevalence Ratio; CI, Confidence Interval; MV, Multivariable

[a]Age (continuous), race, stage, insurance, and urban/rural status

Supplementary Table 4. Age- and multivariable-adjusted prevalence ratios for the association between neighborhood deprivation and ODX score, according to stage.

Neighborhood deprivation index	Low-risk ODX Score N (%)	Intermediate-risk ODX Score N (%)	Age-adjusted PR (95% CI)	MV-adjusted PR (95% CI)[a]	High-risk ODX Score N (%)	Age-adjusted PR (95% CI)	MV-adjusted PR (95% CI)[a]
**Stage I patients**							
Quartile 1 (Least deprived)	1844 (60%)	1021 (33%)	Referent	Referent	214 (7%)	Referent	Referent
Quartile 2	1238 (59%)	674 (32%)	1.00 (0.92, 1.08)	1.00 (0.92, 1.08)	184 (9%)	1.26 (1.05, 1.52)	1.16 (0.96, 1.41)
Quartile 3	955 (59%)	519 (32%)	1.00 (0.91, 1.08)	1.00 (0.91, 1.09)	138 (9%)	1.23 (1.01, 1.51)	1.05 (0.84, 1.31)
Quartile 4 (Most deprived)	633 (62%)	301 (29%)	0.91 (0.82, 1.02)	0.90 (0.80, 1.01)	90 (9%)	1.22 (0.97, 1.54)	0.95 (0.74, 1.23)
**Stage II-III patients**							
Quartile 1 (Least deprived)	698 (53%)	470 (36%)	Referent	Referent	144 (11%)	Referent	Referent
Quartile 2	535 (53%)	314 (31%)	0.92 (0.82, 1.03)	0.90 (0.80, 1.01)	154 (15%)	1.32 (1.08, 1.62)	1.20 (0.98, 1.48)
Quartile 3	399 (52%)	270 (35%)	1.01 (0.90, 1.14)	0.97 (0.85, 1.10)	92 (12%)	1.13 (0.90, 1.44)	0.94 (0.73, 1.20)
Quartile 4 (Most deprived)	249 (51%)	154 (31%)	0.96 (0.83, 1.11)	0.90 (0.77, 1.05)	87 (18%)	1.57 (1.24, 1.98)	1.27 (0.98, 1.65)

ODX, Oncotype DX; PR, Prevalence Ratio; CI, Confidence Interval; MV, Multivariable

[a]Age (continuous), race, stage, insurance, and urban/rural status

Supplementary Table 5. Distribution of 11,377 non-Hispanic Black (NHB) and White (NHW) women diagnosed with stage I-IIIa breast cancer in Georgia with Oncotype Dx Recurrence Scores (ODX) and registered with the Georgia Cancer Registry by TAILORx groups

Characteristic	Overall, N = 11,377	Q1 (Least deprived), N = 4,391	Q2, N = 3,099	Q3, N = 2,373	Q4 (Most deprived), N = 1,514
ODX TAILORx groups, n (%)					
RS 0-25	9,463 (83%)	3,725 (85%)	2,540 (82%)	1,963 (83%)	1,235 (82%)
RS 26+	1,881 (17%)	653 (15%)	550 (18%)	403 (17%)	275 (18%)

NOTE: Unknown values were not included in the calculation of percentages

Supplementary Table 6: Age- and multivariable-adjusted prevalence ratios (PR) for the association between neighborhood deprivation and TAILORx ODX Score High-risk vs. Low-risk for each neighborhood quartile

Neighborhood deprivation index	Low-risk ODX Score N (%)	High-risk ODX Score N (%)	Age-adjusted PR (95% CI)	MV-adjusted PR (95% CI)[a]
Quartile 1 (Least deprived)	3725 (85%)	653 (15%)	Referent	Referent
Quartile 2	2540 (82%)	550 (18%)	1.20 (1.08, 1.33)	1.12 (1.01, 1.25)
Quartile 3	1963 (83%)	403 (17%)	1.15 (1.03, 1.29)	1.04 (0.92, 1.17)
Quartile 4 (Most deprived)	1235 (82%)	275 (18%)	1.24 (1.09, 1.41)	1.05 (0.92, 1.21)

ODX, Oncotype DX; PR, Prevalence Ratio; CI, Confidence Interval; MV, Multivariable
[a]Age, Race, Stage, Insurance Status, and Urban/Rural status.

Supplementary Table 7: Age-and multivariable-adjusted prevalence ratios for the association between neighborhood deprivation and TAILORx ODX score, according to race

Neighborhood deprivation index	Low-risk ODX Score N (%)	High-risk ODX Score N (%)	Age-adjusted PR (95% CI)	MV-adjusted PR (95% CI)[a]
**Non-Hispanic Black patients**				
Quartile 1 (Least deprived)	377 (77%)	114 (23%)	Referent	Referent
Quartile 2	569 (77%)	168 (23%)	0.98 (0.80, 1.21)	1.01 (0.82, 1.24)
Quartile 3	609 (78%)	167 (22%)	0.96 (0.78, 1.18)	0.99 (0.80, 1.23)
Quartile 4 (Most deprived)	608 (80%)	154 (20%)	0.93 (0.75, 1.15)	0.99 (0.80, 1.23)
**Non-Hispanic White patients**				
Quartile 1 (Least deprived)	3348 (86%)	539 (14%)	Referent	Referent
Quartile 2	1971 (84%)	382 (16%)	1.17 (1.04, 1.32)	1.12 (0.99, 1.27)
Quartile 3	1354 (85%)	236 (15%)	1.07 (0.93, 1.23)	1.02 (0.88, 1.19)
Quartile 4 (Most deprived)	627 (84%)	121 (16%)	1.16 (0.97, 1.39)	1.11 (0.91, 1.34)

ODX, Oncotype DX; PR, Prevalence Ratio; CI, Confidence Interval; MV, Multivariable

[a]Age, Stage, Insurance Status, and Urban/Rural status.
